# 1-Acetyl-5-isobutyl-2-sulfanylidene­imidazolidin-4-one

**DOI:** 10.1107/S1600536810037256

**Published:** 2010-09-30

**Authors:** Kaozhen Li, Qiu Feng, Ze-Hua Lu

**Affiliations:** a College of Chemistry and Chemical Engineering, Liaocheng University, Shandong 252059, People’s Republic of China; b Liaocheng International Peace Hospital, Shandong 252059, People’s Republic of China

## Abstract

There are two independent mol­ecules in the asymmetric unit of the title compound, C_9_H_14_N_2_O_2_S. In the crystal, the mol­ecules are linked by N—H⋯O hydrogen bonds, forming a chain along the *a* axis.

## Related literature

For biological activity of thio­hydantoins, see: Lopez & Trigo (1985 ▶). For a related structure, see: Sulbaran *et al.* (2007 ▶).
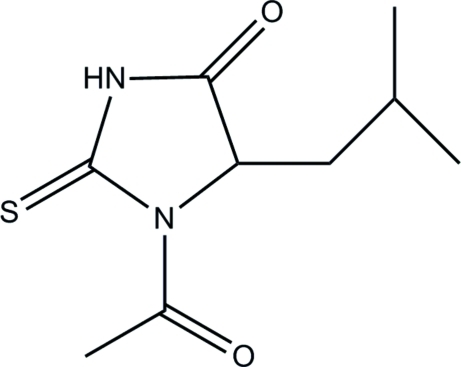


## Experimental

### 

#### Crystal data

C_9_H_14_N_2_O_2_S
*M* *_r_* = 214.28
Triclinic, 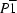

*a* = 7.1856 (8) Å
*b* = 9.7567 (11) Å
*c* = 16.4226 (15) Å
α = 101.231 (2)°
β = 93.965 (1)°
γ = 90.491 (1)°
*V* = 1126.3 (2) Å^3^
*Z* = 4
Mo *K*α radiation
μ = 0.27 mm^−1^
*T* = 298 K
0.42 × 0.35 × 0.14 mm

#### Data collection

Bruker SMART APEX CCD area-detector diffractometer
Absorption correction: multi-scan (*SADABS*; Sheldrick, 1996 ▶) *T*_min_ = 0.897, *T*_max_ = 0.964
5877 measured reflections
3916 independent reflections
2199 reflections with *I* > 2σ(*I*)
*R*_int_ = 0.023

#### Refinement

*R*[*F* ^2^ > 2σ(*F* ^2^)] = 0.054
*wR*(*F* ^2^) = 0.138
*S* = 1.02
3916 reflections
259 parameters
H-atom parameters constrained
Δρ_max_ = 0.27 e Å^−3^
Δρ_min_ = −0.17 e Å^−3^

### 

Data collection: *SMART* (Siemens, 1996 ▶); cell refinement: *SAINT* (Siemens, 1996 ▶); data reduction: *SAINT*; program(s) used to solve structure: *SHELXS97* (Sheldrick, 2008 ▶); program(s) used to refine structure: *SHELXL97* (Sheldrick, 2008 ▶); molecular graphics: *SHELXTL* (Sheldrick, 2008 ▶); software used to prepare material for publication: *SHELXTL*.

## Supplementary Material

Crystal structure: contains datablocks I, global. DOI: 10.1107/S1600536810037256/is2592sup1.cif

Structure factors: contains datablocks I. DOI: 10.1107/S1600536810037256/is2592Isup2.hkl

Additional supplementary materials:  crystallographic information; 3D view; checkCIF report

## Figures and Tables

**Table 1:** Hydrogen-bond geometry (Å, °)

*D*—H⋯*A*	*D*—H	H⋯*A*	*D*⋯*A*	*D*—H⋯*A*
N2—H2⋯O2^i^	0.86	1.99	2.834 (3)	167
N4—H4⋯O4^ii^	0.86	2.01	2.850 (3)	167

Symmetry codes: (i) 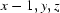; (ii) 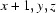.

**Fig. 1.**
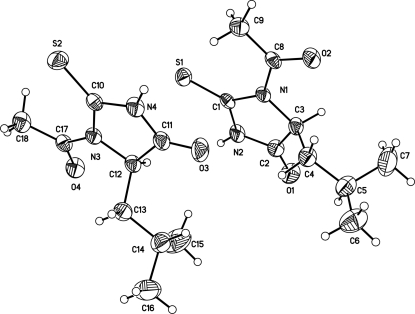
The content of asymmetric unit of the title compound showing the atomic numbering scheme and 30% probability displacement ellipsoids.

## supplementary crystallographic information

### Comment

Thiohydantoins are known to exhibit a wide range of biological activities, including anticonvulsant, antiarrhythmic, anti-inflammatory, and antidiabetic properties, as well as herbicidal and fungicidal activity (Lopez & Trigo, 1985).

In the title compound (Fig. 1), the bond lengths and angles are normal and comparable to those observed in the reported compound (Sulbaran *et al.*, 2007). In the crystal structure, there exist intermolecular N—H···O hydrogen bonds (Table 1). The supramolecular chain structure along the *a* axis, which is built by weak intermolecular N—H···O hydrogen bonds.

### Experimental

2-Amino-4-methylpentanoic acid (10 mmol) and NH_4_SCN (10 mmol) was dissolved in a 15 ml acetic anhydride and 2 ml acetic acid mixture and transferred in a round-bottom flask. The mixture was warmed, with agitation, to 373 K over a period of 1 h. The resulting solution was cooled in a ice/water mixture and stored in a freezer overnight. Crystal of (I) suitable for X-ray diffraction analysis were obtained by slow evaporation. Elemental analysis: calculated for C_9_H_14_N_2_O_2_S: C 50.45, H 6.59, N 13.07%; found: C 50.53, H 6.46, N 13.14%.

### Refinement

All H atoms were placed in geometrically idealized positions (N—H 0.86 and C—H 0.96–0.97 Å) and treated as riding on their parent atoms, with *U*_iso_(H) = 1.2*U*_eq_(C, N) or 1.5*U*_eq_(methyl C).

### Crystal data

**Table:**

C_9_H_14_N_2_O_2_S	*Z* = 4
*M**_r_* = 214.28	*F*(000) = 456
Triclinic, *P*1	*D*_x_ = 1.264 Mg m^−^^3^
Hall symbol: -P 1	Mo *K*α radiation, λ = 0.71073 Å
*a* = 7.1856 (8) Å	Cell parameters from 1409 reflections
*b* = 9.7567 (11) Å	θ = 2.5–22.5°
*c* = 16.4226 (15) Å	µ = 0.27 mm^−^^1^
α = 101.231 (2)°	*T* = 298 K
β = 93.965 (1)°	Block, colourless
γ = 90.491 (1)°	0.42 × 0.35 × 0.14 mm
*V* = 1126.3 (2) Å^3^	

### Data collection

**Table:**

Bruker SMART APEX CCD area-detector diffractometer	3916 independent reflections
Radiation source: fine-focus sealed tube	2199 reflections with *I* > 2σ(*I*)
graphite	*R*_int_ = 0.023
φ and ω scans	θ_max_ = 25.0°, θ_min_ = 2.1°
Absorption correction: multi-scan (*SADABS*; Sheldrick, 1996)	*h* = −8→8
*T*_min_ = 0.897, *T*_max_ = 0.964	*k* = −9→11
5877 measured reflections	*l* = −18→19

### Refinement

**Table:**

Refinement on *F*^2^	Primary atom site location: structure-invariant direct methods
Least-squares matrix: full	Secondary atom site location: difference Fourier map
*R*[*F*^2^ > 2σ(*F*^2^)] = 0.054	Hydrogen site location: inferred from neighbouring sites
*wR*(*F*^2^) = 0.138	H-atom parameters constrained
*S* = 1.02	*w* = 1/[σ^2^(*F*_o_^2^) + (0.0539*P*)^2^ + 0.2948*P*] where *P* = (*F*_o_^2^ + 2*F*_c_^2^)/3
3916 reflections	(Δ/σ)_max_ = 0.001
259 parameters	Δρ_max_ = 0.27 e Å^−^^3^
0 restraints	Δρ_min_ = −0.17 e Å^−^^3^

### Special details

**Table:**

Geometry. All e.s.d.'s (except the e.s.d. in the dihedral angle between two l.s. planes) are estimated using the full covariance matrix. The cell e.s.d.'s are taken into account individually in the estimation of e.s.d.'s in distances, angles and torsion angles; correlations between e.s.d.'s in cell parameters are only used when they are defined by crystal symmetry. An approximate (isotropic) treatment of cell e.s.d.'s is used for estimating e.s.d.'s involving l.s. planes.
Refinement. Refinement of *F*^2^ against ALL reflections. The weighted *R*-factor *wR* and goodness of fit *S* are based on *F*^2^, conventional *R*-factors *R* are based on *F*, with *F* set to zero for negative *F*^2^. The threshold expression of *F*^2^ > σ(*F*^2^) is used only for calculating *R*-factors(gt) *etc*. and is not relevant to the choice of reflections for refinement. *R*-factors based on *F*^2^ are statistically about twice as large as those based on *F*, and *R*- factors based on ALL data will be even larger.

### Fractional atomic coordinates and isotropic or equivalent isotropic displacement parameters (Å^2^)

**Table:**

	*x*	*y*	*z*	*U*_iso_*/*U*_eq_	
N1	0.3760 (3)	0.2881 (3)	0.16750 (16)	0.0404 (7)	
N2	0.0770 (3)	0.2612 (3)	0.18054 (17)	0.0523 (8)	
H2	−0.0417	0.2710	0.1746	0.063*	
N3	0.0219 (3)	0.7835 (3)	0.17203 (16)	0.0429 (7)	
N4	0.3235 (4)	0.7558 (3)	0.18537 (17)	0.0518 (8)	
H4	0.4406	0.7664	0.1800	0.062*	
O1	0.0752 (3)	0.1110 (3)	0.27212 (17)	0.0746 (8)	
O2	0.6815 (3)	0.2536 (3)	0.16880 (16)	0.0638 (7)	
O3	0.3503 (3)	0.6072 (3)	0.27747 (17)	0.0748 (8)	
O4	−0.2837 (3)	0.7469 (3)	0.17042 (16)	0.0649 (7)	
S1	0.13550 (12)	0.41653 (10)	0.06933 (6)	0.0563 (3)	
S2	0.23535 (13)	0.90538 (11)	0.07109 (6)	0.0626 (3)	
C1	0.2002 (4)	0.3238 (3)	0.13812 (19)	0.0403 (8)	
C2	0.1575 (4)	0.1820 (4)	0.2329 (2)	0.0520 (9)	
C3	0.3646 (4)	0.2041 (4)	0.2328 (2)	0.0466 (9)	
H3	0.4243	0.1141	0.2158	0.056*	
C4	0.4495 (5)	0.2774 (4)	0.3174 (2)	0.0569 (10)	
H4A	0.5733	0.3121	0.3106	0.068*	
H4B	0.3747	0.3576	0.3373	0.068*	
C5	0.4652 (6)	0.1881 (4)	0.3833 (2)	0.0698 (11)	
H5	0.3394	0.1543	0.3900	0.084*	
C6	0.5376 (7)	0.2777 (5)	0.4657 (3)	0.1015 (16)	
H6A	0.6642	0.3068	0.4621	0.152*	
H6B	0.5328	0.2245	0.5089	0.152*	
H6C	0.4616	0.3585	0.4782	0.152*	
C7	0.5853 (8)	0.0619 (5)	0.3603 (3)	0.121 (2)	
H7A	0.7066	0.0916	0.3486	0.182*	
H7B	0.5283	0.0007	0.3119	0.182*	
H7C	0.5972	0.0131	0.4058	0.182*	
C8	0.5509 (4)	0.3120 (3)	0.1399 (2)	0.0453 (8)	
C9	0.5730 (5)	0.4020 (4)	0.0785 (2)	0.0639 (11)	
H9A	0.7025	0.4090	0.0686	0.096*	
H9B	0.5277	0.4934	0.0996	0.096*	
H9C	0.5030	0.3621	0.0273	0.096*	
C10	0.1898 (4)	0.8166 (3)	0.1424 (2)	0.0427 (8)	
C11	0.2580 (5)	0.6769 (4)	0.2376 (2)	0.0502 (9)	
C12	0.0504 (4)	0.6996 (3)	0.2371 (2)	0.0461 (9)	
H12	−0.0175	0.6099	0.2202	0.055*	
C13	−0.0073 (5)	0.7752 (4)	0.3214 (2)	0.0573 (10)	
H13A	0.0745	0.8564	0.3406	0.069*	
H13B	−0.1329	0.8086	0.3142	0.069*	
C14	−0.0024 (6)	0.6886 (4)	0.3882 (2)	0.0724 (12)	
H14	0.1255	0.6567	0.3955	0.087*	
C15	−0.1284 (9)	0.5618 (6)	0.3658 (3)	0.126 (2)	
H15A	−0.1293	0.5155	0.4122	0.190*	
H15B	−0.0840	0.4992	0.3189	0.190*	
H15C	−0.2526	0.5894	0.3518	0.190*	
C16	−0.0487 (7)	0.7818 (6)	0.4705 (3)	0.1089 (17)	
H16A	−0.1747	0.8127	0.4655	0.163*	
H16B	0.0352	0.8614	0.4831	0.163*	
H16C	−0.0355	0.7296	0.5143	0.163*	
C17	−0.1606 (4)	0.8094 (4)	0.1444 (2)	0.0471 (9)	
C18	−0.2005 (5)	0.9099 (4)	0.0897 (2)	0.0630 (10)	
H18A	−0.3330	0.9169	0.0800	0.094*	
H18B	−0.1466	0.8784	0.0376	0.094*	
H18C	−0.1478	0.9998	0.1157	0.094*	

### Atomic displacement parameters (Å^2^)

**Table:**

	*U*^11^	*U*^22^	*U*^33^	*U*^12^	*U*^13^	*U*^23^
N1	0.0242 (14)	0.0472 (17)	0.0518 (16)	0.0029 (11)	0.0067 (12)	0.0132 (14)
N2	0.0207 (14)	0.067 (2)	0.074 (2)	0.0038 (13)	0.0043 (13)	0.0246 (17)
N3	0.0261 (14)	0.0508 (17)	0.0527 (17)	0.0051 (12)	0.0052 (12)	0.0115 (15)
N4	0.0248 (15)	0.0608 (19)	0.074 (2)	0.0033 (13)	0.0075 (14)	0.0212 (17)
O1	0.0495 (16)	0.092 (2)	0.094 (2)	−0.0114 (14)	0.0122 (14)	0.0441 (18)
O2	0.0275 (13)	0.0854 (19)	0.0865 (18)	0.0089 (12)	0.0085 (12)	0.0342 (16)
O3	0.0490 (16)	0.089 (2)	0.098 (2)	0.0208 (14)	0.0066 (14)	0.0459 (18)
O4	0.0317 (13)	0.0777 (18)	0.0915 (19)	0.0010 (12)	0.0099 (13)	0.0299 (16)
S1	0.0400 (5)	0.0709 (7)	0.0607 (6)	0.0080 (4)	−0.0020 (4)	0.0209 (5)
S2	0.0469 (6)	0.0798 (8)	0.0682 (7)	0.0025 (5)	0.0149 (5)	0.0280 (6)
C1	0.0262 (17)	0.044 (2)	0.048 (2)	0.0048 (14)	0.0041 (14)	0.0028 (17)
C2	0.0330 (19)	0.062 (2)	0.065 (2)	−0.0020 (17)	0.0063 (17)	0.020 (2)
C3	0.0324 (18)	0.051 (2)	0.060 (2)	0.0032 (15)	0.0030 (16)	0.0207 (19)
C4	0.044 (2)	0.064 (2)	0.064 (2)	−0.0014 (18)	0.0012 (18)	0.019 (2)
C5	0.064 (3)	0.085 (3)	0.064 (3)	−0.005 (2)	−0.001 (2)	0.026 (2)
C6	0.104 (4)	0.130 (4)	0.068 (3)	−0.016 (3)	−0.008 (3)	0.021 (3)
C7	0.152 (5)	0.109 (4)	0.106 (4)	0.043 (4)	−0.021 (4)	0.037 (4)
C8	0.0260 (18)	0.051 (2)	0.060 (2)	0.0049 (15)	0.0078 (16)	0.0102 (19)
C9	0.042 (2)	0.073 (3)	0.085 (3)	0.0027 (19)	0.0208 (19)	0.031 (2)
C10	0.0293 (18)	0.045 (2)	0.052 (2)	0.0036 (15)	0.0036 (15)	0.0055 (17)
C11	0.036 (2)	0.055 (2)	0.062 (2)	0.0085 (17)	0.0075 (17)	0.016 (2)
C12	0.0333 (18)	0.049 (2)	0.058 (2)	0.0047 (15)	0.0077 (16)	0.0141 (19)
C13	0.049 (2)	0.064 (2)	0.062 (2)	0.0079 (18)	0.0116 (18)	0.016 (2)
C14	0.067 (3)	0.085 (3)	0.072 (3)	0.008 (2)	0.011 (2)	0.028 (3)
C15	0.173 (6)	0.110 (4)	0.108 (4)	−0.037 (4)	0.031 (4)	0.044 (4)
C16	0.115 (4)	0.143 (5)	0.070 (3)	0.022 (4)	0.023 (3)	0.020 (3)
C17	0.0322 (19)	0.051 (2)	0.058 (2)	0.0043 (16)	0.0054 (16)	0.0091 (19)
C18	0.040 (2)	0.072 (3)	0.079 (3)	0.0074 (18)	−0.0009 (19)	0.023 (2)

### Geometric parameters (Å, °)

**Table:**

N1—C1	1.393 (4)	C6—H6B	0.9600
N1—C8	1.398 (4)	C6—H6C	0.9600
N1—C3	1.478 (4)	C7—H7A	0.9600
N2—C2	1.368 (4)	C7—H7B	0.9600
N2—C1	1.372 (4)	C7—H7C	0.9600
N2—H2	0.8600	C8—C9	1.476 (5)
N3—C10	1.390 (4)	C9—H9A	0.9600
N3—C17	1.399 (4)	C9—H9B	0.9600
N3—C12	1.472 (4)	C9—H9C	0.9600
N4—C10	1.361 (4)	C11—C12	1.510 (4)
N4—C11	1.363 (4)	C12—C13	1.524 (4)
N4—H4	0.8600	C12—H12	0.9800
O1—C2	1.209 (4)	C13—C14	1.508 (5)
O2—C8	1.218 (4)	C13—H13A	0.9700
O3—C11	1.205 (4)	C13—H13B	0.9700
O4—C17	1.217 (4)	C14—C15	1.497 (6)
S1—C1	1.626 (3)	C14—C16	1.534 (5)
S2—C10	1.635 (3)	C14—H14	0.9800
C2—C3	1.502 (4)	C15—H15A	0.9600
C3—C4	1.517 (5)	C15—H15B	0.9600
C3—H3	0.9800	C15—H15C	0.9600
C4—C5	1.515 (5)	C16—H16A	0.9600
C4—H4A	0.9700	C16—H16B	0.9600
C4—H4B	0.9700	C16—H16C	0.9600
C5—C7	1.512 (6)	C17—C18	1.471 (5)
C5—C6	1.517 (5)	C18—H18A	0.9600
C5—H5	0.9800	C18—H18B	0.9600
C6—H6A	0.9600	C18—H18C	0.9600
			
C1—N1—C8	129.4 (3)	C8—C9—H9A	109.5
C1—N1—C3	111.9 (2)	C8—C9—H9B	109.5
C8—N1—C3	118.6 (3)	H9A—C9—H9B	109.5
C2—N2—C1	114.9 (3)	C8—C9—H9C	109.5
C2—N2—H2	122.6	H9A—C9—H9C	109.5
C1—N2—H2	122.6	H9B—C9—H9C	109.5
C10—N3—C17	129.3 (3)	N4—C10—N3	105.3 (3)
C10—N3—C12	111.7 (2)	N4—C10—S2	123.4 (2)
C17—N3—C12	118.8 (3)	N3—C10—S2	131.3 (3)
C10—N4—C11	115.1 (3)	O3—C11—N4	126.2 (3)
C10—N4—H4	122.5	O3—C11—C12	127.9 (3)
C11—N4—H4	122.5	N4—C11—C12	105.9 (3)
N2—C1—N1	104.9 (3)	N3—C12—C11	101.5 (3)
N2—C1—S1	123.4 (2)	N3—C12—C13	112.7 (3)
N1—C1—S1	131.7 (2)	C11—C12—C13	112.0 (3)
O1—C2—N2	125.9 (3)	N3—C12—H12	110.1
O1—C2—C3	127.8 (3)	C11—C12—H12	110.1
N2—C2—C3	106.3 (3)	C13—C12—H12	110.1
N1—C3—C2	101.6 (2)	C14—C13—C12	115.1 (3)
N1—C3—C4	113.5 (3)	C14—C13—H13A	108.5
C2—C3—C4	111.9 (3)	C12—C13—H13A	108.5
N1—C3—H3	109.9	C14—C13—H13B	108.5
C2—C3—H3	109.9	C12—C13—H13B	108.5
C4—C3—H3	109.9	H13A—C13—H13B	107.5
C5—C4—C3	115.3 (3)	C15—C14—C13	112.9 (4)
C5—C4—H4A	108.5	C15—C14—C16	111.7 (4)
C3—C4—H4A	108.5	C13—C14—C16	108.9 (4)
C5—C4—H4B	108.5	C15—C14—H14	107.7
C3—C4—H4B	108.5	C13—C14—H14	107.7
H4A—C4—H4B	107.5	C16—C14—H14	107.7
C7—C5—C4	113.2 (4)	C14—C15—H15A	109.5
C7—C5—C6	110.9 (4)	C14—C15—H15B	109.5
C4—C5—C6	109.3 (3)	H15A—C15—H15B	109.5
C7—C5—H5	107.7	C14—C15—H15C	109.5
C4—C5—H5	107.7	H15A—C15—H15C	109.5
C6—C5—H5	107.7	H15B—C15—H15C	109.5
C5—C6—H6A	109.5	C14—C16—H16A	109.5
C5—C6—H6B	109.5	C14—C16—H16B	109.5
H6A—C6—H6B	109.5	H16A—C16—H16B	109.5
C5—C6—H6C	109.5	C14—C16—H16C	109.5
H6A—C6—H6C	109.5	H16A—C16—H16C	109.5
H6B—C6—H6C	109.5	H16B—C16—H16C	109.5
C5—C7—H7A	109.5	O4—C17—N3	115.9 (3)
C5—C7—H7B	109.5	O4—C17—C18	122.3 (3)
H7A—C7—H7B	109.5	N3—C17—C18	121.7 (3)
C5—C7—H7C	109.5	C17—C18—H18A	109.5
H7A—C7—H7C	109.5	C17—C18—H18B	109.5
H7B—C7—H7C	109.5	H18A—C18—H18B	109.5
O2—C8—N1	116.2 (3)	C17—C18—H18C	109.5
O2—C8—C9	122.6 (3)	H18A—C18—H18C	109.5
N1—C8—C9	121.1 (3)	H18B—C18—H18C	109.5

### Hydrogen-bond geometry (Å, °)

**Table:**

*D*—H···*A*	*D*—H	H···*A*	*D*···*A*	*D*—H···*A*
N2—H2···O2^i^	0.86	1.99	2.834 (3)	167
N4—H4···O4^ii^	0.86	2.01	2.850 (3)	167

Symmetry codes: (i) *x*−1, *y*, *z*; (ii) *x*+1, *y*, *z*.
